# Depression, Anxiety and Glucose Metabolism in the General Dutch Population: The New Hoorn Study

**DOI:** 10.1371/journal.pone.0009971

**Published:** 2010-04-01

**Authors:** Vanessa Bouwman, Marcel C. Adriaanse, Esther van ’t Riet, Frank J. Snoek, Jacqueline M. Dekker, Giel Nijpels

**Affiliations:** 1 Department of Prevention and Public Health, Faculty of Earth and Life Sciences, VU University Amsterdam, Amsterdam, The Netherlands; 2 EMGO Institute for Health and Care Research, VU University Medical Center, Amsterdam, The Netherlands; 3 Department of Epidemiology and Biostatistics, VU University Medical Center, Amsterdam, The Netherlands; 4 Department of Medical Psychology, VU University Medical Center, Amsterdam, The Netherlands; 5 Department of General Practice, VU University Medical Center, Amsterdam, The Netherlands; University of Wuerzburg, Germany

## Abstract

**Background:**

There is a well recognized association between depression and diabetes. However, there is little empirical data about the prevalence of depressive symptoms and anxiety among different groups of glucose metabolism in population based samples. The aim of this study was to determine whether the prevalence of increased levels of depression and anxiety is different between patients with type 2 diabetes and subjects with impaired glucose metabolism (IGM) and normal glucose metabolism (NGM).

**Methodology/Principal Findings:**

Cross-sectional data from a population-based cohort study of 2667 residents, 1261 men and 1406 women aged 40–65 years from the Hoorn region, the Netherlands. Depressive symptoms and anxiety were measured using the Centre for Epidemiologic Studies Depression Scale (CES-D, score ≥16) and the Hospital Anxiety and Depression Scale – Anxiety Subscale (HADS-A, score ≥8), respectively. Glucose metabolism status was determined by oral glucose tolerance test. In the total study population the prevalence of depressive symptoms and anxiety for the NGM, IGM and type 2 diabetes were 12.5, 12.2 and 21.0% (P = 0.004) and 15.0, 15.3 and 19.9% (p = 0.216), respectively. In men, the prevalence of depressive symptoms was 7.7, 9.5 and 19.6% (p<0.001), and in women 16.4, 15.8 and 22.6 (p = 0.318), for participants with NGM, IGM and type 2 diabetes, respectively. Anxiety was not associated with glucose metabolism when stratified for sex. Intergroup differences (NGM vs. IGM and IGM vs. type 2 diabetes) revealed that higher prevalences of depressive symptoms are mainly manifested in participants with type 2 diabetes, and not in participants with IGM.

**Conclusions:**

Depressive symptoms, but not anxiety are associated with glucose metabolism. This association is mainly determined by a higher prevalence of depressive symptoms in participants with type 2 diabetes and not in participants with IGM.

## Introduction

There is a well recognized association between depression and diabetes. Evidence suggests that the relationship between these conditions is bi-directional [1]. Yet, recently it was concluded that there is a strong and robust association between depression and the incidence of type 2 diabetes, but only a weak relation between diabetes and the risk of depression [2]. The underlying mediating factors and mechanisms of these relationships remain to be elucidated.

It is well-known that patients with depression and diabetes, compared with patients with diabetes alone, have been linked with poor self-care and adherence to medical treatment [3], poorer glycemic control [4], more diabetes complications [5], and increased health-care use and costs [6]. Moreover, depression in diabetes patients is associated with a higher risk of morbidity and all cause mortality [7].

Only a few studies have addressed the prevalence of depressive symptoms among different groups of glucose metabolism, especially in persons with impaired glucose metabolism, or pre-diabetes, with conflicting findings. To our knowledge Knol et al [8] were the first reporting that in contrast to diagnosed type 2 diabetes, impaired fasting glucose was not associated with depressive symptoms. We previously observed a gender difference, where depressive symptoms were found to be more common in women with impaired glucose metabolism, but not in men [9]. Other studies have shown evidence that psychological distress increased the risk of pre-diabetes in young Swedish subjects [10], no association between depressive symptoms and unrecognized glucose intolerance in the United States adults [11], and a gender-specific association in elderly men but not women living in the United Kingdom [12]. No association was found between fasting glucose and depression among middle-aged men [13]. Recently Aujla et al [14] concluded that depression was not significantly more prevalent in people with type 2 diabetes and impaired glucose metabolism in a multi-ethnic population. Other important variations such as study design, type of population studied, depression measures used, and definition of glucose status used have led to these discrepancies. We suggested that previous findings needed to be enlightened to further disentangle these associations, with additional attention to the role of anxiety. So far there has been less focus on anxiety in patients with diabetes. Yet, there is evidence suggesting that anxiety may play a role in the onset of type 2 diabetes [15].

Thus, the current study expands on this prior work by investigating whether the prevalence of increased levels of depression and anxiety is different between patients with type 2 diabetes and subjects with impaired glucose metabolism (IGM) and normal glucose metabolism (NGM). For this purpose we used cross sectional data from The New Hoorn Study, a large population based-cohort study.

## Methods

### Participants

The study has been conducted according to the principles expressed in the Declaration of Helsinki. All participants provided written informed consent of their willingness to be involved. The study was approved by the Medical Ethics Committee of the VU University Medical Center Amsterdam.

From July 2006 until November 2007, a population-based study on glucose tolerance was performed in the city of Hoorn, the Netherlands (The New Hoorn Study). The New Hoorn Study has been described in detail previously [16]. In short, a random sample of 6180 men and women aged 40–65 years was drawn from the municipal population registry of Hoorn. Participants were invited to visit the Diabetes Research Center in Hoorn. Of this sample, 2807 people agreed to participate (45.4%). Of these, 12 participants were excluded because of missing questionnaire data, 76 participants were excluded because they had >4 items missing on the CES-D or >1 item on the HADS-A, and another 52 participants were excluded because of missing glucose tolerance status. The remaining 2667 participants constituted the sample for the current study. The final response rate is 2667/6180 (43%).

### Depressive symptoms

Depressive symptoms were measured using the validated Dutch version of the 20-item Centre for Epidemiologic Studies Depression Scale (CES-D) [17]. The CES-D measures the frequency of depressive symptoms over the past 7 days. The CES-D consists of 20 items and the total score can range from 0–60. The generally used cut-off score of 16 and above was used to identify respondents with clinically significant levels of depressive symptoms. In participants with ≤4 items missing, scores were imputed by person mean score.

### Anxiety

Anxiety was measured using the Dutch version of the validated Hospital Anxiety and Depression Scale – Anxiety Subscale (HADS-A) [18]. The HADS-A is a scale originally developed to indicate the possible presence of anxiety in the setting of a medical outpatient clinic. Nowadays it is also used to indicate the presence of anxiety in general medical patients [19]. The HADS-A consists of 7 items and the total score can range from 0 to 21. The generally used cut-off score of 8 and above was used to identify respondents with the possible presence of anxiety [20]. In participants with 1 item missing, the score was imputed by person mean score.

### Socio-demographic and clinical outcomes

Data were collected during the medical examination that was carried out at the Diabetes Research Centre in Hoorn. Information about age, sex, smoking, family history of diabetes and education was assessed by means of a questionnaire. Smoking status was defined into current smokers, past-smokers and never-smokers. Education was classified into low, middle and high educational level.

Fasting whole blood glucose from a capillary vein in the finger was determined on the spot using a HemoCue Beta-glucose analyzer (HemoCue). In 2641 of the 2667 participants, who had a fasting whole blood glucose level below 10 mmol/l, a standard 75-g oral glucose tolerance test (OGTT) was performed. Glucose was measured in venous plasma by the glucose-oxidase method (Glucoquant/hexokinase/G6P-DH; Boehringer-Mannheim, Mannheim, Germany). Glucose metabolism status was defined according to the World Health Organization 2006 criteria [21].; people with normal glucose metabolism (NGM), with impaired glucose metabolisms (IGM) and with type 2 diabetes NGM was defined as fasting plasma glucose (FPG) <6.1 mmol/l and 2 hr plasma glucose (2hrPG) <7.8 mmol/l. IGM was defined as FPG 6.1–6.9 mmol/l or 2 hr PG 7.8–11.0 mmol/l. Type 2 diabetes was defined as FPG ≥7.0 mmol/l or 2hrPG ≥11.1 mmol/l. HbA1c was assessed using a DCCT standardized reversed-phase cation exchange chromatography (HA 8160 analyzer, Menarini, Florence, Italy) Triglycerides, HDL-cholesterol and total cholesterol were determined from fasting plasma samples by enzymatic techniques (Boehringer-Mannheim, Mannheim, Germany).

The waist circumference was measured between the lower rib margin and the spina iliaca anterior superior. Blood pressure was measured 3 times on the right arm after a 10 minute rest period, using a Colin Press BP 8800p Non-Invasive Blood Pressure Monitor (Colin Medical Technology Coorporation, USA). Final blood pressure was calculated as the mean of the last 2 measurements. Participants were considered to be hypertensive if their systolic pressure was >140 and/or if the diastolic pressure was >90 and/or if they were taking antihypertensive medication.

### Statistical analysis

Descriptive data are presented for the three groups of glucose metabolism status, i.e., NGM, IGM and type 2 diabetes. Differences in study sample characteristics for the three groups of glucose metabolism status were examined using analysis of variance (ANOVA) for continue variables and χ^2^-tests for dichotomous and categorical variables. Differences in both CES-D (score ≥16) and HADS-A (score≥8) prevalences and mean depressive symptoms and anxiety scores by glucose metabolism were calculated for the total sample and for men and women separately, using χ^2^-tests and ANOVA’s respectively. Additional intergroup analyses were performed to test differences in prevalence and mean scores of depressive symptoms and anxiety comparing NGM vs. IGM and IGM vs. type 2 diabetes in the total sample and stratified by sex.

Next, logistic regression analyses were carried out to contrast the data on depressive symptoms (CES-D score ≥16) and anxiety (score≥8), comparing subjects with IGM or type 2 diabetes with NGM subjects. In analyses pertaining to depressive symptoms and anxiety, unadjusted and adjusted odds ratios with 95% confidence intervals were obtained separately for men and women. We tested whether adjustment for demographic variables (age, education and family history of diabetes) and cardiovascular risk factors (triglycerides, HDL cholesterol, total cholesterol, waist circumference, hypertension and smoking), changed the association of glucose status with depression and anxiety.

To further analyse the association of depression with type 2 diabetes subjects and IGM, logistic regression was performed with depression as the outcome, and type 2 diabetes and IGM as predictor variables. The odds ratios and 95% confidence intervals of the unadjusted models (type 2 diabetes vs. NGM and IGM vs. NGM) and adjusted models (age, education, family history of diabetes, triglycerides, HDL cholesterol, total cholesterol, waist circumference, hypertension and smoking) were calculated. Pearson’s correlations were applied to determine the association between continuous CES-D and HADS-A scores. All analyses were performed using SPSS (version 15.0). For all statistical testing, we used two-sided hypothesis testing. The p-value for statistical significance was set at ≤0.05.

## Results

### Study population

Descriptive statistics of the total study population are presented in table 1. The total study sample consisted of 2667 participants, 1261 men (47.3%) and 1406 women, with a mean age of 53.4 years (SD±6.7). Of those 832 participants were aged 40 to 49 (31.2%), 1242 participants were aged 50 to 59 (46.6%) and 593 participants were aged 60 to 66 (22.2%).

**Table 1:** Descriptive statistics of the total study population.

Male (N (%))	1261 (47.3)	
Low education (N (%))	673 (25.7)	
Current smoking (N (%))	566 (21.4)	
Family history of diabetes (N (%))	734 (27.7)	
Glucose metabolism status (N (%))		
Type 2 diabetes	181 (6.8)	
Impaired glucose metabolism	425 (15.9)	
Normal glucose metabolism	2061 (77.3)	
Age (years)	N = 2667	53.4±6.7
Fasting Plasma glucose (mmol/l)	N = 2766	5.6±1.0
2-hour postload plasma glucose	N = 2641	5.9±2.4
HbA1c (%)	N = 2663	5.5±0.5
Triglycerides (mmol/l)	N = 2664	1.5±1.1
HDL cholesterol (mmol/l)	N = 2662	1.5±0.4
Total cholesterol mmol/l)	N = 2664	5.5±1.0
Waist circumference (cm)	N = 2665	89.8±11.8
Hypertension (N (%))	870 (32.7)	
Depression prevalence (N (% CES-D ≥16)	348 (13.0)	
Depression (CES-D mean ± SD)	N = 2667	7.4±7.8
Anxiety prevalence (N (% HADS-A ≥8))	410 (15.4)	
Anxiety (HADS-A mean ± SD)	N = 2667	4.2±3.5

Data are numbers (N), %, means ± SD. Abbreviations: HADS-A, Hospital Anxiety and Depression Scale – Anxiety Subscale; CES-D, Center for Epidemiologic Studies Depression Scale; SD, standard deviation.

Characteristics of the participants by glucose metabolism status are presented in table 2. NGM was apparent in 2061 (77.3%) participants, 425 (15.9%) had IGM and 181 (6.8%) type 2 diabetes. Age, fasting plasma glucose, 2 hour plasma glucose, HbA1c, triglycerides, HDL-cholesterol, total cholesterol, waist circumference, hypertension and smoking were all significantly associated with glucose metabolism status.

**Table 2:** Characteristics of the study population by glucose metabolism status.

	NGM	IGM	DM2	P
N	2061	425	181	
Male (%)	44.8	56.7	53.6	<0.001
Age (years)	52.8±6.7	55.2±6.2	56.3±6.1	<0.001
Low education (%)	24.4	30.0	30.4	0.080
Current smoking (%)	21.0	22.9	21.1	0.001
Family history of diabetes (%)	25.6	31.9	41.7	<0.001
Fasting Plasma glucose (mmol/l)	5.3±0.4	6.0±0.5	7.8±2.2	<0.001
2-hour postload plasma glucose	5.1±1.1	7.5±1.8	12.1±4.3	<0.001
HbA1c (%)	5.3±0.3	5.6±0.3	6.5±1.3	<0.001
Triglycerides (mmol/l)	1.3±0.7	1.8±1.1	2.4±2.8	<0.001
HDL cholesterol (mmol/l)	1.6±0.4	1.4±0.4	1.3±0.4	<0.001
Total cholesterol mmol/l)	5.4±1.0	5.6±1.1	5.3±1.3	0.023
Waist circumference (cm)	87.7±10.6	95.4±11.8	100.1±13.9	<0.001
Hypertension (%)	32.0	56.7	66.9	<0.001

Data are %, means ± SD. P  =  p-values based on one-way ANOVA for continuous variables and χ^2^-tests for categorical variables. Abbreviations: NGM, normal glucose metabolism; IGM, impaired glucose metabolism; DM2, type 2 diabetes; HbA1c, glycated haemoglobin; HDL, high-density lipoprotein; SD, standard deviation.

### Depressive symptoms and anxiety in the total study population

The prevalence (% CES-D score ≥16 and HADS-A score ≥8) and mean scores of depressive symptoms and anxiety by glucose metabolism status for the total study population and stratified by sex are presented in Table 3. In the total population 229 out of 2667 (8.6%) subjects suffered from both anxiety and depression (158 female (11%) and 71 male (5.6%)). The prevalence of depressive symptoms for participant with NGM, IGM and type 2 diabetes was 12.5, 12.2 and 21.0% (P<0.05), respectively. Higher mean depression scores were significantly associated with worse metabolic status (P<0.01). Regarding anxiety, both the prevalences (% HADS-A score ≥8; NGM: 15.0, IGM: 15.3 and type 2 diabetes: 19.9%; P = 0.216) and mean scores (NGM: 4.2±3.5, IGM: 3.9±3.6 and type 2 diabetes: 4.4±3.9; P = 0.196) for the three glucose metabolism groups showed a similar trend as depressive symptoms, but were not significant. CES-D was associated with HADS-A scores (r = 0.69).

**Table 3:** Prevalence and mean scores of depressive symptoms and anxiety by glucose metabolism status for the total study population and stratified according to sex.

	Total				Male				Female			
	NGM (N = 2061)	IGM (N = 425)	DM2 (N = 181)	P	NGM (N = 923)	IGM (N = 241)	DM2 (N = 97)	P	NGM (N = 1138)	IGM (N = 184)	DM2 (N = 84)	P
**CES-D**												
≥16 (%)	12.5	12.2	21.0^a^	0.004	7.7	9.5	19.6^a^	<0.001	16.4	15.8	22.6	0.318
Mean±SD	7.3±7.5	7.0±7.1	9.4±8.9^a^	0.001	6.1±6.6	6.1±6.7	8.4±8.8^a^	0.007	8.3±8.1	8.1±7.5	10.6±9.0^a^	0.043
**HADS-A**												
≥8 (%)	15.0	15.3	19.9	0.216	10.2	11.6	16.5	0.155	18.9	20.1	23.8	0.524
Mean±SD	4.2±3.5	3.9±3.6	4.4±3.9	0.196	3.7±3.2	3.5±3.4	4.0±3.7	0.389	4.6±3.6	4.5±3.8	4.9±3.7	0.689

Data are %, means ± SD. P  =  p-values based on one-way ANOVA for continuous variables and χ^2^-tests for categorical variables. ^a^ significantly different comparing IGM vs. DM2. Abbreviations: NGM, normal glucose metabolism; IGM, impaired glucose metabolism; DM2, type 2 diabetes; SD, standard deviation; HADS-A. Hospital Anxiety and Depression Scale – Anxiety Subscale; CES-D, Center for Epidemiologic Studies Depression Scale.

To further analyse the influence of possible confounders on the association of depression with type 2 diabetes subjects and IGM, logistic regression was performed with depression as the outcome, and type 2 diabetes and IGM as predictor variables (Table 4). When comparing DM2 with IGM the unadjusted odds ratio 1.86 (CI 95% 1.27–2.72) slightly dropped to 1.77 (CI 95% 1.13–2.78) after full adjustment of age, sex, education, family history of diabetes, triglycerides, HDL cholesterol, total cholesterol, hypertension, smoking and waist circumference.

**Table 4:** Logistic regression with depression (CES-D ≥16) as the outcome, inclusive of unadjusted models and models adjusted for confounding factors in the total population.

	Odds Ratio (95% CI)	P-value
*Model 1(unadjusted)*		
IGM vs. NGM*	0.97 (0.71–1.34)	0.872
DM2 vs. NGM*	1.86 (1.27–2.72)	0.001
*Model 2*		
IGM vs. NGM*	1.13 (0.81–1.57)	0.461
DM2 vs. NGM*	2.02 (1.35–3.04)	0.001
Middle/high vs. low education*	0.70 (0.53–0.94)	0.017
Female vs. Male*	1.91 (1.50–2.45)	<0.001
Age	0.98 (0.96–0.99)	0.007
Family history of DM2 vs. no family history of DM2*	1.10 (0.85–1.42)	0.476
*Model 3*		
IGM vs. NGM*	1.09 (0.78–1.54)	0.643
DM2 vs. NGM*	1.77 (1.13–2.78)	0.013
Middle/high vs. low education*	0.71 (0.53–0.95)	0.022
Female vs. Male*	2.10 (1.59–2.78)	<0.001
Age	0.99 (0.97–1.00)	0.104
Family history of DM2 vs. no family history of DM2*	1.09 (0.84–1.41)	0.526
Triglycerides	1.21 (1.02–1.43)	0.027
HDL cholesterol	1.17 (0.82–1.67)	0.402
Total cholesterol	0.80 (0.70–0.92)	0.002
Hypertension vs. no hypertension*	0.59 (0.44–0.79)	<0.001
Current smoker vs. non-current smoker*	1.21 (0.92–1.60)	0.177
Waist circumference	1.01 (1.00–1.02)	0.096

Abbreviations: NGM, normal glucose metabolism; IGM, impaired glucose metabolism; DM2, type 2 diabetes (WHO 2006 criteria).

*Denotes reference category.

### Depressive symptoms and anxiety by sex

The prevalence and mean scores of both depressive symptoms and anxiety differ by sex (data not shown). In men, the prevalences of depressive symptoms in the groups with NGM, IGM and type 2 diabetes were 7.7, 9.5 and 19.6% (P<0.001), respectively. Mean depressive symptoms scores for NGM (6.1±6.6), IGM (6.1±6.7) and type 2 diabetes (8.4±8.8), confirmed the significant (P<0.010) relationship between depressive symptoms and glucose metabolism in men. This relationship could not be confirmed for anxiety.

In women with NGM, IGM and type 2 diabetes the prevalences of depressive symptoms were 16.8, 15.8 and 22.6% (P = 0.318) and the mean depressive symptoms scores were 8.3 (±8.1), 8.1 (±7.5) and 10.6% (±9.0) (P = 0.043), respectively. The prevalence of anxiety (P = 0.542) and the mean anxiety scores (P = 0.689) were again not significantly associated with glucose metabolism in women.

Logistic regression analyses with depression (CES-D score ≥16) and anxiety (score≥8) by sex with IGM and type 2 diabetes subjects compared with NGM subjects were performed, including unadjusted and adjusted models (Table 5). The unadjusted odds ratio of 2.92 (CI 95% 1.68–5.10) for depression in men decreased 2.55 CI 95% 1.34–4.86) after adjustment for age, education, family history of diabetes, triglycerides, HDL cholesterol, total cholesterol, hypertension, smoking and waist circumference, though remain significant. In women, none of the odds ratios for depression in the unadjusted and adjusted models (Model 1, 2 and 3) were significant.

**Table 5:** Odds ratios for depression (CES-D score ≥16) and anxiety (HADS-A ≥16) by sex with impaired glucose metabolism or type 2 diabetes subjects compared with normal glucose metabolism subjects.

		n	%	Unadjusted OR (95 CI)*	Model 1 OR (95 CI)§	Model 2 OR (95 CI)¶	Model 3 OR (95 CI)†
**Depression**							
*Men*	NGM	923	7.7	referent	referent	referent	referent
	IGM	241	9.5	1.27 (0.77–2.07)	1.26 (0.76–2.09)	1.13 (0.67–1.90)	1.14 (0.67–1.94)
	DM2	97	19.6	2.92 (1.68–5.10)	3.16 (1.78–5.62)	2.55 (1.38–4.73)	2.55 (1.34–4.86)
*Women*	NGM	1138	16.4	referent	referent	referent	referent
	IGM	184	15.8	0.95 (0.62–1.46)	1.07 (0.69–1.66)	1.08 (0.66–1.63)	1.05 (0.67–1.67)
	DM2	84	22.6	1.49 (0.78–2.54)	1.39 (0.78–2.49)	1.30 (0.70–2.40)	1.37 (0.71–2.62)
**Anxiety**							
*Men*	NGM	923	10.2	referent	referent	referent	referent
	IGM	241	11.6	1.16 (0.74–1.81)	1.11 (0.70–1.78)	1.08 (0.67–1.75)	1.07 (0.65–1.76)
	DM2	97	16.5	1.74 (0.98–3.10)	1.91 (1.06–3.47)	1.73 (0.92–3.26)	1.58 (0.82–3.06)
*Women*	NGM	1138	18.9	referent	referent	referent	referent
	IGM	184	20.1	1.08 (0.73–1.60)	1.16 (0.78–1.74)	1.12 (0.74–1.70)	1.14 (0.74–1.75)
	DM2	84	23.8	1.34 (0.80–2.27)	1.33 (0.76–2.31)	1.24 (0.69–2.23)	1.36 (0.74–2.51)

Abbreviations: NGM, normal glucose metabolism; IGM, impaired glucose metabolism; DM2, type 2 diabetes (WHO 2006 criteria).

* The unadjusted odds ratios.

§ Model 1: Adjusted for age, education and family history of diabetes.

¶ Model 2: Adjusted for Model 1 and triglycerides, HDL cholesterol, and total cholesterol.

† Model 3. Adjusted for Model 2 and, hypertension, smoking and waist circumference.

### Intergroup differences

Additional analyses were performed to test intergroup differences (NGM vs. IGM and IGM vs. type 2 diabetes) in prevalence and mean scores of depressive symptoms and anxiety by glucose metabolism status for the total sample and by sex (Table 3). We found no differences in the prevalences of depression and mean depressive symptoms when comparing NGM versus IGM neither for the total population nor for men or women separately. However, when comparing IGM versus type 2 diabetes, all prevalences of depression and mean depressive symptoms scores for the total sample and across sex appeared to be different, except for the depressive symptoms prevalences among women (P = 0.174). No differences in prevalences of anxiety and mean anxiety scores for the total sample and by sex were found comparing NGM vs. IGM and IGM vs. type 2 diabetes. These results strongly suggests that the association between depressive symptoms and glucose metabolism is mainly determined by the higher prevalence of depressive symptoms in participants with type 2 diabetes and not in participants with IGM.

## Discussion

To our knowledge, this is the first study inquiring both depressive symptoms and anxiety among different groups of glucose metabolism status in a large population based sample using oral glucose tolerance test. Our results show an association between depressive symptoms and glucose metabolism status but not anxiety. In addition, this association seems to be mainly determined by the higher prevalence of depressive symptoms in participants with type 2 diabetes and not in participants with IGM. This finding is in line with previous studies [8], [10], [12], although other studies did not show an association [11]. The outcomes partly match with previous findings of our study group [9]. We previously concluded that depressive symptoms were more common in women with impaired glucose metabolism. This conclusion is not fully supported by our present study. This could be due to differences in sample size, methodological issues and age of the studied population. Given the large sample size of our present study population and scoring patterns of the primary outcomes across glucose metabolism, the total sample and sex, it is reasonable to believe that the findings presented are valid. Our results do not confirm the results of a study by Aujla et. al., concluding that depression was not significantly more prevalent in people with type 2 diabetes and impaired glucose metabolism [14]. A possible explanation is the use of different measures in defining depression. Where Aujla et. al., defined depression by a WHO-5 wellbeing score ≤13, we used a depression specific instrument (CES-D), using the well documented cut-score of ≥16.

Based on the work of Engum [15], and the marked degree of correlation between anxiety and depressive symptoms in our sample, we expected to find a relation between anxiety and glucose metabolism status. However, our data did not show such a relationship. Moreover, we found no association between anxiety and glucose metabolism in neither the total sample or in men and women separately. These findings are robust and appeared to be valid for both the prevalence (dichotomous) and mean (continuous) anxiety scores. Yet, the outcomes need to be interpreted with caution since anxiety scores show virtually the same distributions among the three glucose groups as depression scores, though not significant. The fact that we did not find such a relationship is hard to explain, but could be attributed to the difference in anxiety measure at baseline (Anxiety and Depression Index) and definition of diabetes (self-reported) by Engum. Further prospective and experimental studies are needed to disentangle the role of depression and anxiety (generalized anxiety, panic disorder, phobias) in relation to glucose metabolism.

Impaired glucose metabolism is an important predictor of developing type 2 diabetes [22] and, a bidirectional relationship between depressive symptoms and glucose metabolism status exists [1]. This implies that depressive symptoms would gradually rise among the three different groups of glucose metabolism. Our results do not support this hypothesis. We found highest levels of depressive symptoms in subjects with type 2 diabetes and not in NGM or IGM. This pattern was true for the total study sample as well as for men and women separately. Inter group comparisons confirmed these findings.

In this study highest levels of depressive symptoms in subjects with type 2 diabetes were found. It has been suggested that the onset of depressive symptoms comes from the stresses and strains of the knowledge that one has type 2 diabetes [23]. However, this is unlikely given the accumulating evidence that newly diagnosed type 2 diabetes has no substantial short and long term psychological impact [24].

The mechanisms that underlie the association between depression and type 2 diabetes are unclear, though have been described previously [9], [15]. Potential mechanistic pathways include the influence of depressive symptoms on behavioural factors, such as sedentary lifestyles, smoking and overeating, resulting is metabolic disturbances, which may explain the onset of diabetes. Alternative mechanisms include, for example, deregulation of the hypothalamic–pituitary–adrenal axis and the sympathetic nervous system [25], sex steroid hormone levels [26], low grade inflammation [27], or vitamin D deficiency [28]. More research is needed with regard to factors underlying these biological mechanisms.

The strengths of our study are that we used data from a large population-based sample of male and female subjects. Gold-standard assessment was used to determine glucose metabolism status (i.e., OGTT). Next, we used the CES-D and HADS-A which are both validated and widely used instruments including well-documented cut-points, also applied in populations with diabetes [29]. Finally, the outcomes are presented for the total population and for men and women separately because of the significant difference in primary outcomes by sex.

Some limitations of our study need to be addressed. First, the present study has a cross-sectional design, thus we cannot infer causality between depressive symptoms, anxiety and glucose metabolism status. Second, we used a self-report measure (CES-D) to measure depressive symptoms, while the psychiatric diagnostic interview is considered the gold standard. However this diagnostic interview is not easy to apply in large-scale epidemiological studies. Third, the excluded participants were more often female and more often low educated. It is possible that the percentage of women with depressive symptoms in our study population is under- or overestimated. However, this excluded proportion of participants is only a small percentage of the total population and therefore not likely to influence the outcomes. Finally, the present study was limited to a Dutch Caucasian adult population. It is not clear whether the relationship between depression, anxiety and glucose is consistent across different ethnic and age groups. Interestingly, no differences in prevalent depression were found in a multi-ethnic population recently [14].

In conclusion, the present study shows in a large population based sample that depressive but not anxiety is associated with glucose metabolism status. This association is mainly determined by a higher prevalence of depressive symptoms in participants with type 2 diabetes and not in participants with IGM. Further well-designed prospective research is needed: (a) to test whether depression and or anxiety, using the psychiatric diagnostic interview, is associated with an increased risk of developing type 2 diabetes, diagnosed according to an OGTT; (b) to disentangle the complex causal relationships between (the onset of) type 2 diabetes, depressive symptoms and anxiety; and (c) to clarify pathophysiological mechanisms underlying these relationships.
